# Incidence of cassava mosaic disease and associated whitefly vectors in South West and North Central Nigeria: Data exploration

**DOI:** 10.1016/j.dib.2018.05.016

**Published:** 2018-05-19

**Authors:** Angela O. Eni, Oghenevwairhe P. Efekemo, Mojisola G. Soluade, Segun I. Popoola, Aderemi A. Atayero

**Affiliations:** a Department of Biological Sciences, Covenant University, Ota, Nigeria; b West African Virus Epidemiology (WAVE) for root and tuber crops, Covenant University Hub, Ota, Nigeria; c Department of Electrical and Information Engineering, Covenant University, Ota, Nigeria; d IoT-Enabled Smart and Connected Communities (SmartCU) Research Cluster, Covenant University, Ota, Nigeria

**Keywords:** Cassava mosaic disease, Whitefly vector, Zero hunger, Cassava mosaic virus

## Abstract

Cassava mosaic disease (CMD) is one of the most economically important viral diseases of cassava, an important staple food for over 800 million people in the tropics. Although several *Cassava mosaic virus* species associated with CMD have been isolated and characterized over the years, several new super virulent strains of these viruses have evolved due to genetic recombination between diverse species. In this data article, field survey data collected from 184 cassava farms in 12 South Western and North Central States of Nigeria in 2015 are presented and extensively explored. In each State, one cassava farm was randomly selected as the first farm and subsequent farms were selected at 10 km intervals, except in locations were cassava farms are sporadically located. In each selected farm, 30 cassava plants were sampled along two diagonals and all selected plant was scored for the presence or absence of CMD symptoms. Cassava mosaic disease incidence and associated whitefly vectors in South West and North Central Nigeria are explored using relevant descriptive statistics, box plots, bar charts, line graphs, and pie charts. In addition, correlation analysis, Analysis of Variance (ANOVA), and multiple comparison post-hoc tests are performed to understand the relationship between the numbers of whiteflies counted, uninfected farms, infected farms, and the mean of symptom severity in and across the States under investigation. The data exploration provided in this data article is considered adequate for objective assessment of the incidence and symptom severity of cassava mosaic disease and associated whitefly vectors in farmers’ fields in these parts of Nigeria where cassava is heavily cultivated.

**Specifications Table**

**Table:** 

Subject area	*Biological Science*
More specific subject area	*Cassava Virus Epidemiology*
Type of data	*Tables, graphs, figures, and spreadsheet file*
How data was acquired	*Cassava farms located along major and intermediate roads in all the State in the South West and North Central Nigeria were surveyed. In each State, one cassava farm was randomly selected as the first farm and subsequent farms were selected at 10 km intervals, except in locations were cassava farms are sporadically located. In each selected farm, 30 cassava plants were sampled along two diagonals and all selected plant was scored for the presence or absence of CMD symptoms.*
Data format	*Raw, analyzed*
Experimental factors	*Field survey data collected from 184 cassava farms in 12 South Western and North Central States of Nigeria in 2015 are presented and extensively explored*
Experimental features	*Cassava mosaic disease incidence and associated whitefly vectors in South West and North Central Nigeria are explored using relevant descriptive statistics, box plots, bar charts, line graphs, and pie charts. In addition, correlation analysis, ANOVA, and multiple comparison post-hoc tests are performed.*
Data source location	*184 cassava farms in 12 South Western and North Central States of Nigeria*
Data accessibility	*A comprehensive dataset is presented in Microsoft Excel spreadsheet and attached to this data article as supplementary material*

**Value of the data**

• In addition to its significance as source of food and animal feed, cassava is increasingly becoming an important raw material for several industries including biofuel producing industries [1], [2]. Therefore, addressing the incidence of cassava mosaic disease and associated whitefly vectors is considered pivotal to the realization of the Sustainable Development Goals (SDGs) numbers 1–3 (i.e. no poverty, zero hunger, and good health and well-being) by 2030 [3], [4].
• Nigeria is the highest producer of cassava globally and the plant is heavily cultivated in the South Western and North Central States of Nigeria [5], [6]. The data provided in this data article will help in tackling the challenges of cassava mosaic disease and associated whitefly vectors in South West and North Central Nigeria. This solution will help the country to harness the potentials of cassava as an important source of foreign exchange.
• The data exploration and the statistical analyses provided in this data article are considered adequate for objective assessment of the incidence and symptom severity of cassava mosaic disease and associated whitefly vectors in farmers’ fields in these parts of Nigeria where cassava is heavily cultivated [7], [8], [9].
• The data presented in this article will encourage reproducible research and open new doors of research collaborations towards finding effective solutions to deal with the evolution of new super virulent strains of cassava mosaic viruses.

## 1 Data

Cassava is a major staple food for millions of people in Nigeria and Africa at large. The plant is drought tolerant, grows in all agro-ecological zones in Nigeria and is one of the highest producing crops in terms of carbohydrate produced per hectare [10]. Beyond its use for food and animal feed, cassava is increasingly becoming a crucial raw material for several industries including biofuel producing industries. Cassava therefore has the potentials to become an important source of foreign exchange for Nigeria which is the highest producer of cassava globally [11]. This important plant is however plagued by several viral diseases which threaten its production and productivity. Cassava mosaic disease (CMD), one of the most economically important cassava virus disease, is wide spread in all areas where cassava is grown [12]. The virus is either seed transmitted or transmitted by whitefly vectors [13]. A diversity of cassava mosaic virus species associated with CMD have been isolated and characterized over the years. However, several new super virulent strains of these viruses have evolved over the years due to genetic recombination between diverse species [14]. This data article seeks to evaluate the incidence and symptom severity of cassava mosaic disease and associated whitefly vectors in farmers’ fields in South West and North Central Nigeria where cassava is heavily cultivated.

## 2 Experimental design, materials and methods

**Fig. 1:**
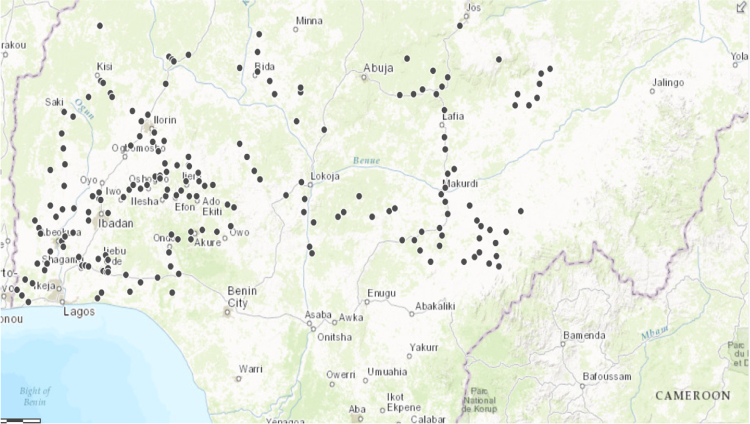
Distribution of 184 cassava farms surveyed in 12 South Western and North Central States of Nigeria in 2015.

Cassava farms located along major and intermediate roads in all the State in the South West and North Central Nigeria were surveyed. The distribution of 184 cassava farms surveyed in 12 South Western and North Central States of Nigeria in 2015 is shown in Fig. 1. In each State, one cassava farm was randomly selected as the first farm and subsequent farms were selected at 10 km intervals except in locations were cassava farms are sporadically located. In each selected farm, 30 cassava plants were sampled along two diagonals and all selected plant was scored for the presence or absence of cassava mosaic disease (CMD) symptoms. Where present, CMD symptom severity was then scored on a scale of 2–5, with 2 indicating mild symptom and 5 indicating very severe symptom covering over 75% of the infected plant. A score of 1 was assigned for none symptomatic plants. The whiteflies present in the top five leaves if each sampled plant were also counted and recorded, to determine the abundance of these CMD vector across the States.

## 3 Data exploration

**Fig. 2:**
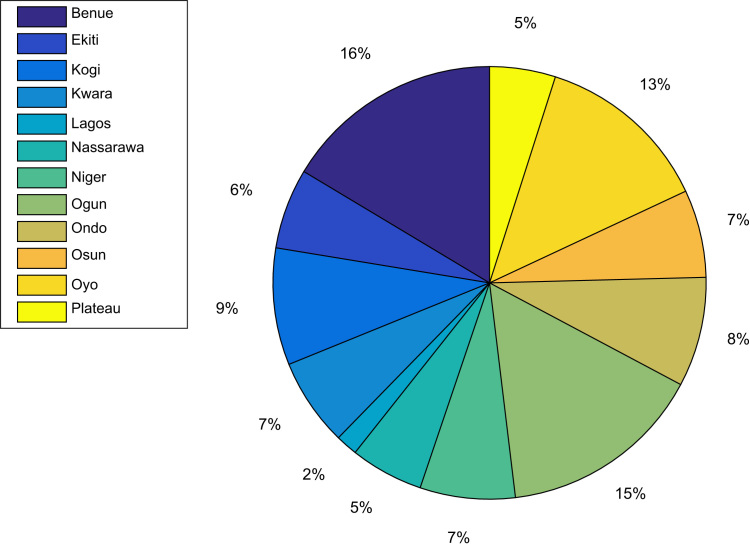
Percentage contribution of each states to the 184 cassava farms covered in this study.

Table 1, Table 2, Table 3, Table 4 present the descriptive statistics (mean, median, mode, standard deviation, variance, kurtosis, Skewness, range, minimum value, maximum value, and the sum) of whiteflies counted, uninfected cassava plants, infected cassava plants, and mean of symptom severity in 184 cassava farms in 12 South Western and North Central States of Nigeria in 2015. The percentage contribution of each of the 12 States is shown in Fig. 2.

**Table 1:** Descriptive statistics of counted whiteflies in 184 farms in 12 Nigerian States.

	Mean	Median	Mode	Standard deviation	Variance	Kurtosis	Skewness	Range	Min	Max	Sum
Benue	0.67	0.00	0	2.19	4.78	18.35	3.96	11	0	11	20
Ekiti	5.45	5.00	0	6.02	36.27	2.13	0.66	17	0	17	60
Kogi	0.00	0.00	0	0.00	0.00	N/A	N/A	0	0	0	0
Kwara	7.75	2.00	0	10.02	100.39	2.84	1.00	30	0	30	93
Lagos	14.67	0.00	0	25.40	645.33	1.50	0.71	44	0	44	44
Nassarawa	0.20	0.00	0	0.42	0.18	3.25	1.50	1	0	1	2
Niger	0.23	0.00	0	0.44	0.19	2.63	1.28	1	0	1	3
Ogun	10.29	1.50	0	16.80	282.29	5.40	1.86	62	0	62	288
Ondo	9.67	7.00	0	9.71	94.38	2.65	0.94	29	0	29	145
Osun	2.75	2.00	0	3.08	9.48	2.40	0.75	9	0	9	33
Oyo	3.67	0.00	0	8.44	71.19	10.21	2.74	36	0	36	88
Plateau	0.00	0.00	0	0.00	0.00	N/A	N/A	0	0	0	0

**Table 2:** Descriptive statistics of uninfected cassava plants cassava plants in 184 farms in 12 Nigerian States.

	Mean	Median	Mode	Standard deviation	Variance	Kurtosis	Skewness	Range	Min	Max	Sum
Benue	12.33	12.50	17	6.09	37.13	3.22	0.42	27	2	29	370
Ekiti	16.55	16.00	12	5.35	28.67	2.11	0.18	18	8	26	182
Kogi	21.19	26.00	28	9.60	92.16	3.08	-1.07	30	0	30	339
Kwara	15.58	14.50	6	7.77	60.45	2.42	0.59	24	6	30	187
Lagos	22.67	22.00	22	1.15	1.33	1.50	0.71	2	22	24	68
Nassarawa	20.90	21.00	20	8.37	70.10	2.53	-0.75	25	5	30	209
Niger	18.69	20.00	30	9.74	94.90	1.62	-0.31	25	5	30	243
Ogun	15.11	14.50	10	6.64	44.03	2.46	0.32	27	3	30	423
Ondo	17.13	16.00	16	7.36	54.12	2.23	0.67	22	8	30	257
Osun	19.33	18.50	15	4.87	23.70	2.46	0.73	15	13	28	232
Oyo	15.25	15.50	1	9.56	91.41	1.86	-0.09	30	0	30	366
Plateau	25.78	30.00	30	6.04	36.44	2.23	-0.95	15	15	30	232

**Table 3:** Descriptive statistics of infected cassava plants.

	Mean	Median	Mode	Standard deviation	Variance	Kurtosis	Skewness	Range	Min	Max	Sum
Benue	17.67	17.50	13	6.09	37.13	3.22	−0.42	27	1	28	530
Ekiti	13.45	14.00	18	5.35	28.67	2.11	−0.18	18	4	22	148
Kogi	8.81	4.00	0	9.60	92.16	3.08	1.07	30	0	30	141
Kwara	14.42	15.50	15	7.77	60.45	2.42	−0.59	24	0	24	173
Lagos	7.33	8.00	8	1.15	1.33	1.50	−0.71	2	6	8	22
Nassarawa	9.10	9.00	10	8.37	70.10	2.53	0.75	25	0	25	91
Niger	11.31	10.00	0	9.74	94.90	1.62	0.31	25	0	25	147
Ogun	14.89	15.50	20	6.64	44.03	2.46	−0.32	27	0	27	417
Ondo	12.87	14.00	0	7.36	54.12	2.23	−0.67	22	0	22	193
Osun	10.67	11.50	2	4.87	23.70	2.46	−0.73	15	2	17	128
Oyo	14.79	14.50	9	9.60	92.09	1.84	0.09	30	0	30	355
Plateau	4.89	0.00	0	6.21	38.61	1.69	0.59	15	0	15	44

Fig. 3, Fig. 4, Fig. 5, Fig. 6, Fig. 7, Fig. 8, Fig. 9, Fig. 10, Fig. 11, Fig. 12, Fig. 13, Fig. 14 give comprehensive information about the whiteflies counted, uninfected cassava plants, infected cassava plants, and mean of symptom severity in 184 cassava farms in Benue, Ekiti, Kogi, Kwara, Lagos, Nassarawa, Niger, Ogun, Ondo, Osun, Oyo, and Plateau States respectively.

**Fig. 3:**
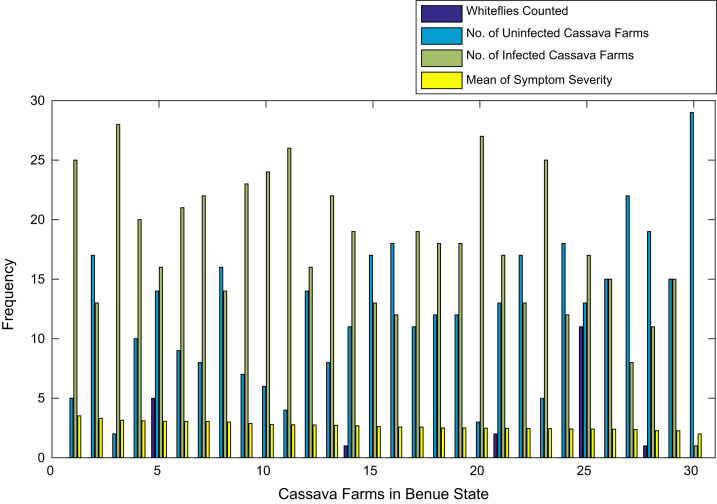
Bar chart showing information about the abundance whiteflies on 30 cassava farms in Benue State.

**Fig. 4:**
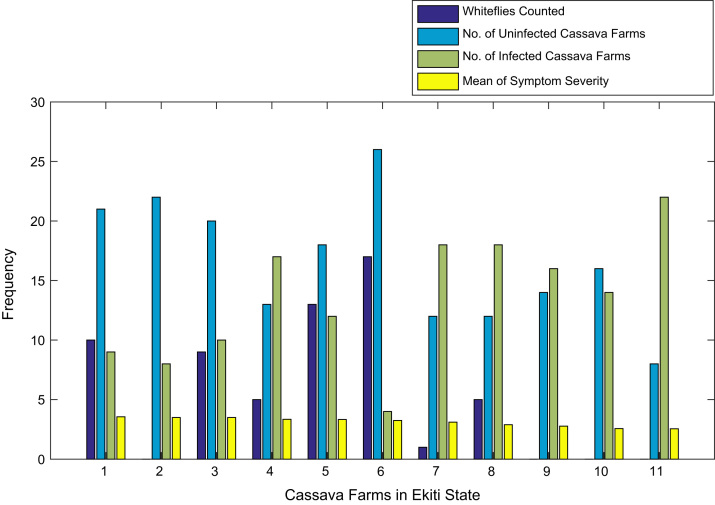
Bar chart showing information about the abundance whiteflies on 11 cassava farms in Ekiti State.

**Fig. 5:**
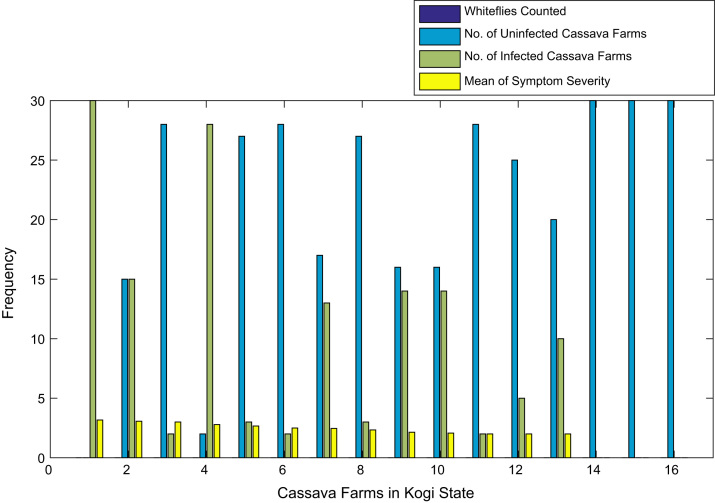
Bar chart showing information the abundance about whiteflies 16 on cassava farms in Kogi State.

**Fig. 6:**
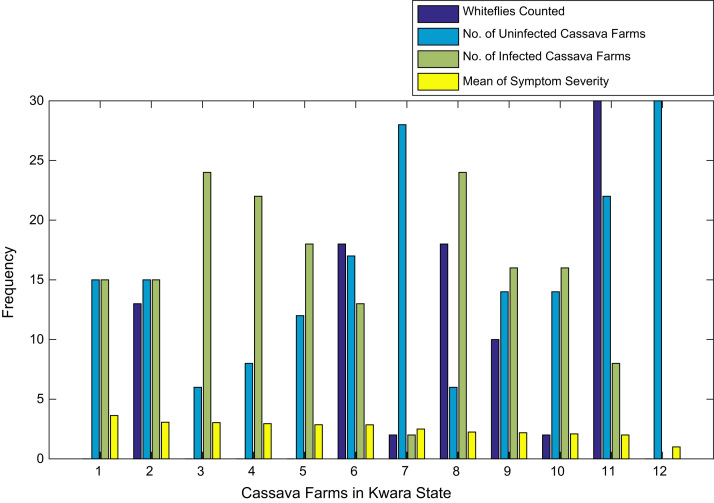
Bar chart showing information the abundance about whiteflies on 12 cassava farms in Kwara State.

**Fig. 7:**
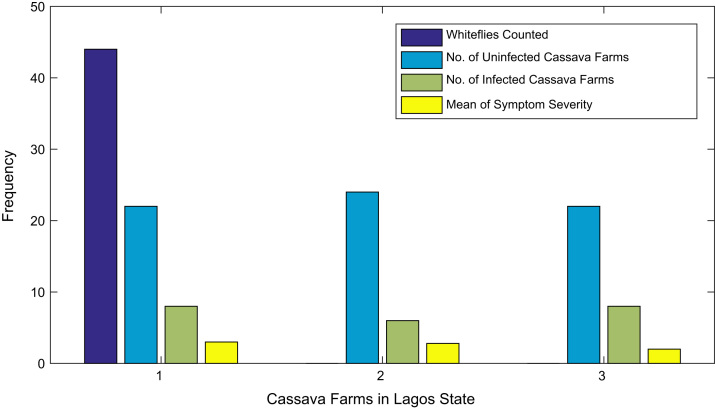
Bar chart showing information the abundance about whiteflies on cassava farms in 3 Lagos State.

**Fig. 8:**
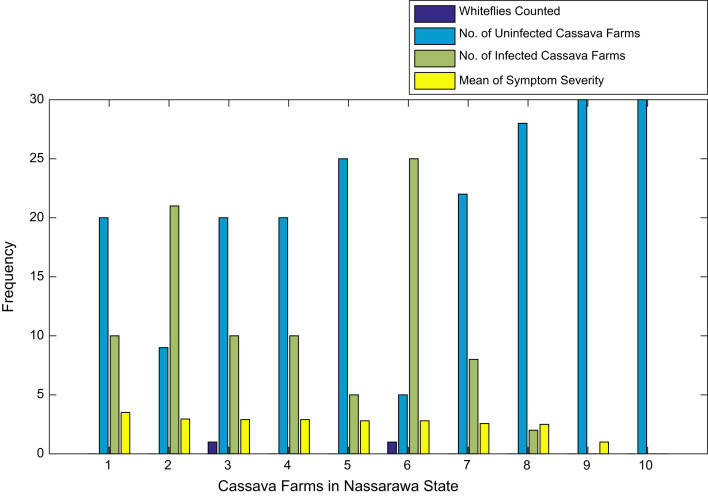
Bar chart showing information about the abundance whiteflies on 10 cassava farms in Nassarawa State.

**Fig. 9:**
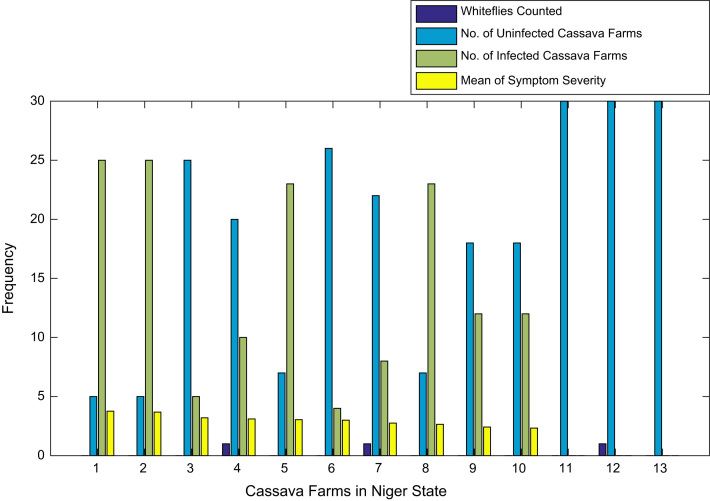
Bar chart showing information about the abundance whiteflies on 13 cassava farms in Niger State.

**Fig. 10:**
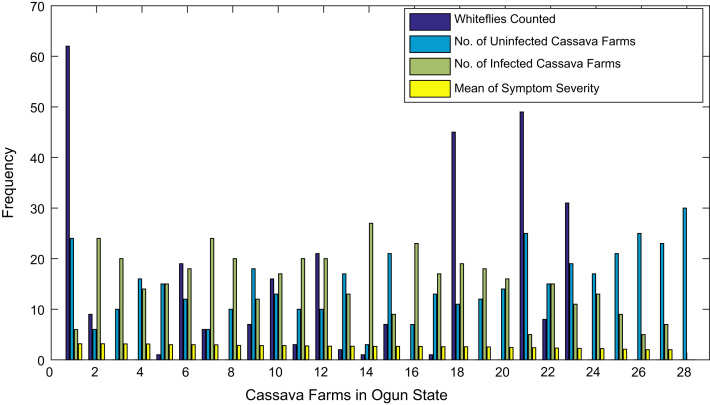
Bar chart showing information about the abundance whiteflies on 28 cassava farms in Ogun State.

**Fig. 11:**
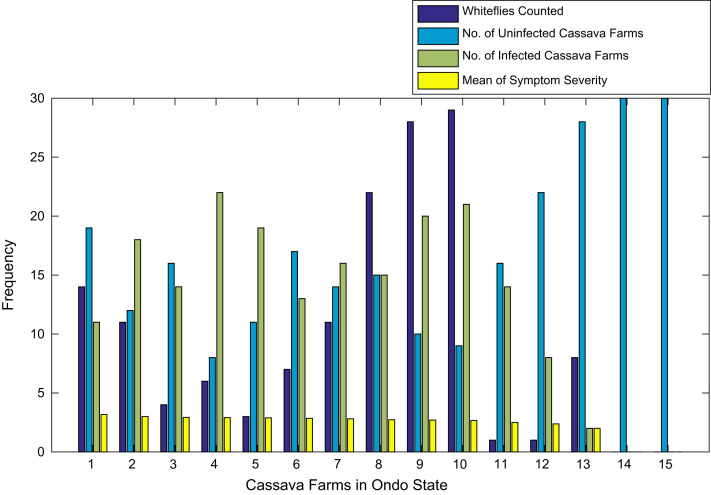
Bar chart showing information about the abundance whiteflies on 15 cassava farms in Ondo State.

**Fig. 12:**
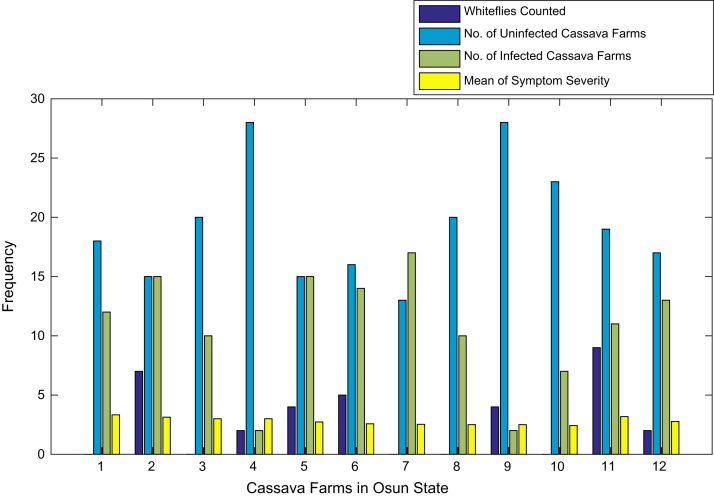
Bar chart showing information about the abundance whiteflies on 12 cassava farms in Osun State.

**Fig. 13:**
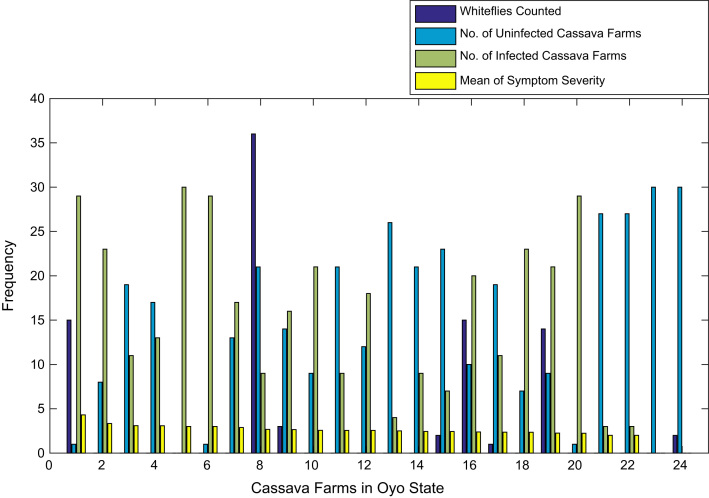
Bar chart showing information about the abundance whiteflies on 24 cassava farms in Oyo State.

**Fig. 14:**
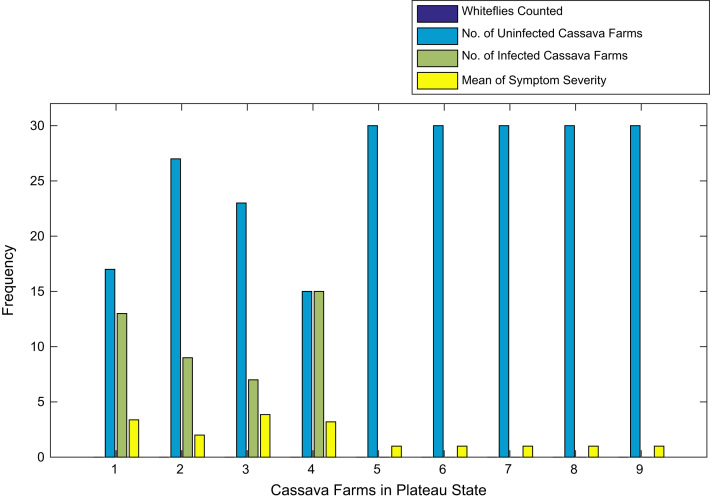
Bar chart showing information about the abundance whiteflies on 9 cassava farms in Plateau State.

**Table 4:** Descriptive statistics of mean of symptom severity.

	Mean	Median	Mode	Standard deviation	Variance	Kurtosis	Skewness	Range	Min	Max	Sum
Benue	2.69	2.60	2.5	0.35	0.12	2.73	0.47	1.52	2	3.52	80.60
Ekiti	3.13	3.25	3.5	0.37	0.14	1.71	-0.42	1.01	2.55	3.56	34.38
Kogi	2.01	2.24	0	1.07	1.14	2.92	-1.11	3.17	0	3.17	32.20
Kwara	2.54	2.68	1	0.68	0.47	3.31	-0.65	2.63	1	3.63	30.43
Lagos	2.60	2.80	2	0.53	0.28	1.50	-0.60	1.00	2	3.00	7.80
Nassarawa	2.39	2.80	2.8	1.06	1.12	3.76	-1.43	3.50	0	3.50	23.92
Niger	2.30	2.75	0	1.38	1.89	2.37	-0.96	3.76	0	3.76	29.93
Ogun	2.56	2.67	2	0.61	0.37	12.37	-2.72	3.17	0	3.17	71.73
Ondo	2.37	2.73	0	1.00	1.00	4.86	-1.84	3.18	0	3.18	35.55
Osun	2.81	2.75	2.5	0.31	0.10	1.65	0.32	0.90	2.43	3.33	33.67
Oyo	2.44	2.53	0	0.90	0.80	5.96	-1.31	4.31	0	4.31	58.65
Plateau	1.94	1.00	1	1.21	1.47	1.58	0.59	2.86	1	3.86	17.44

**Fig. 15:**
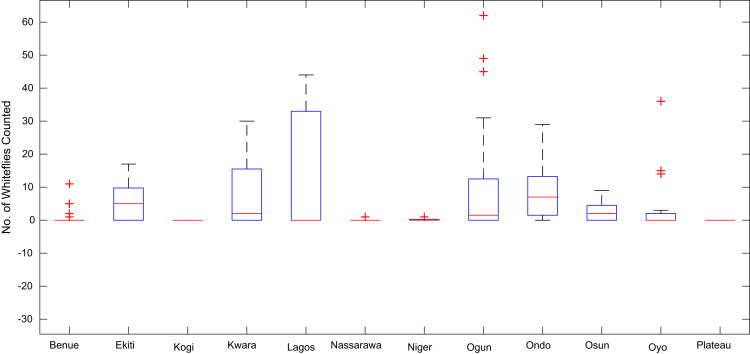
Boxplot representation of no. of whiteflies counted in 184 cassava farms across the 12 Nigerian States.

**Fig. 16:**
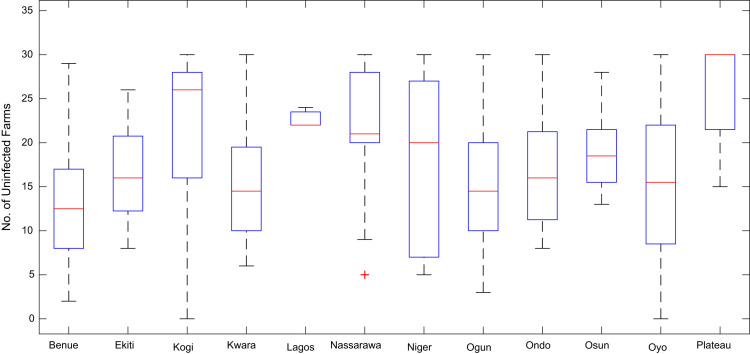
Boxplot representation of no. of uninfected cassava plants in 184 cassava farms sampled across 12 Nigerian States.

**Fig. 17:**
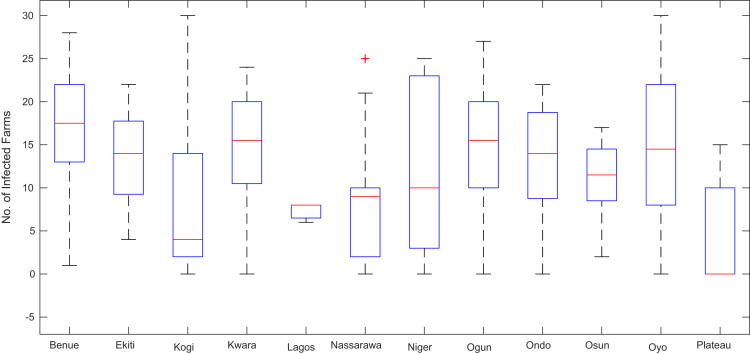
Boxplot representation of no. of infected cassava plants in 184 cassava farms sampled across 12 Nigerian States.

**Fig. 18:**
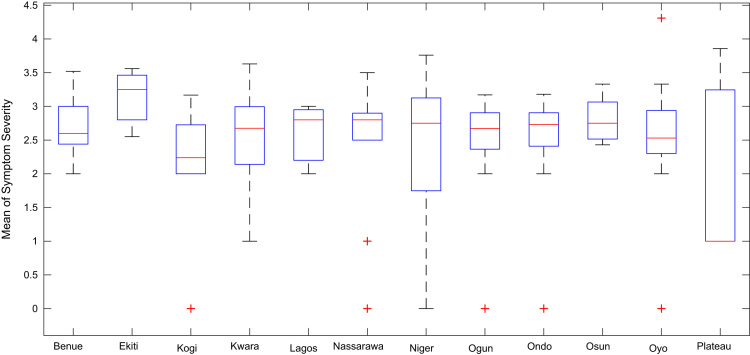
Boxplot representation of mean of Cassava mosaic virus symptom severity across 12 Nigerian States.

Boxplot representations of the numbers of whiteflies counted, uninfected cassava plants, infected cassava plants, and mean of symptom severity in 184 cassava farms across the 12 States of Nigeria are shown in Fig. 15, Fig. 16, Fig. 17, Fig. 18 respectively. The boxplot representations allow visual and statistical comparisons of the data distributions in terms of quartiles.

**Fig. 19:**
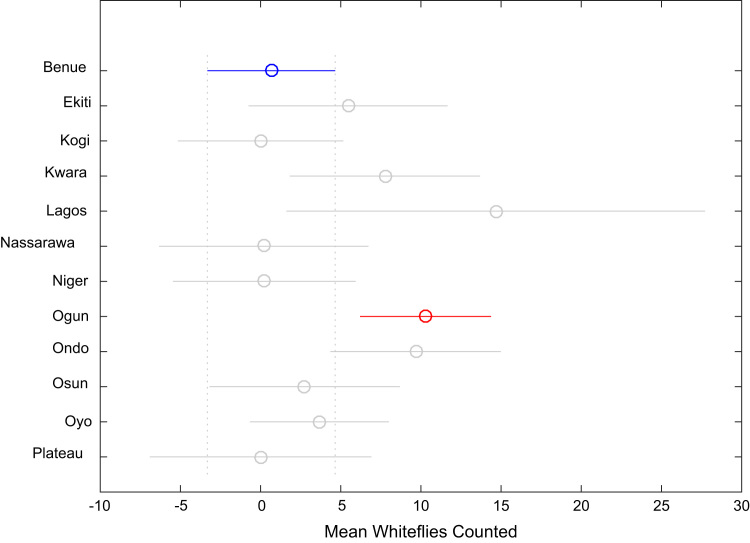
Multiple comparison post-hoc for mean whiteflies counted in 184 farms in 12 Nigerian States.

**Fig. 20:**
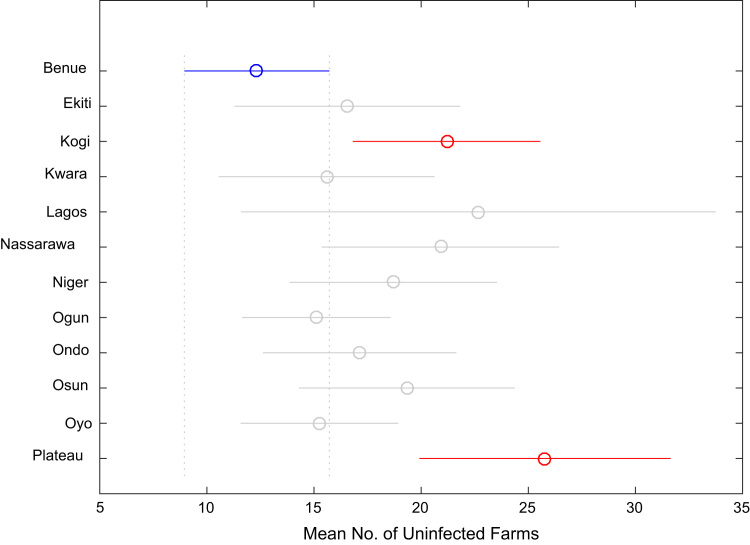
Multiple comparison post-hoc for mean uninfected cassava plants in 184 farms in 12 Nigerian States.

**Fig. 21:**
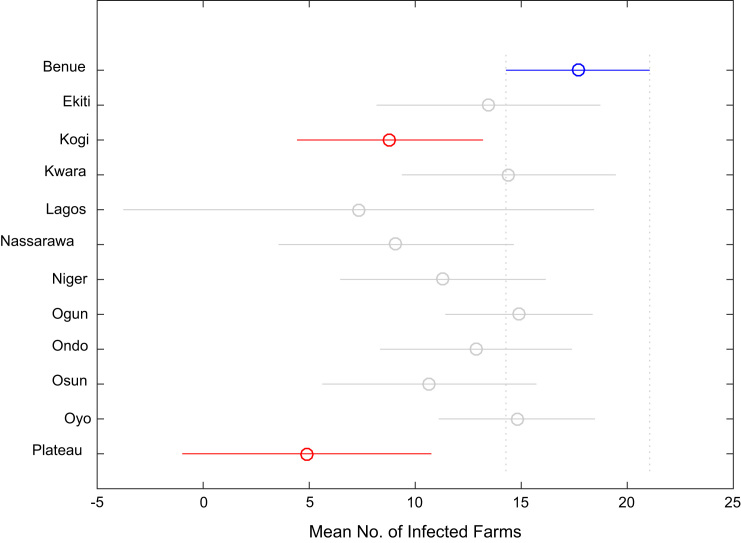
Multiple comparison post-hoc for mean infected cassava plants in 184 farms in 12 Nigerian States.

**Fig. 22:**
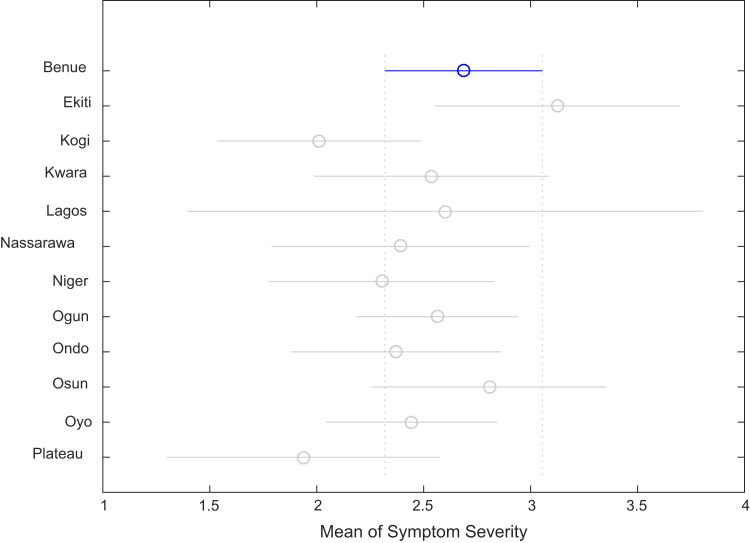
Multiple comparison post-hoc for mean of Cassava mosaic disease symptom severity.

Correlation analysis, ANOVA, and multiple comparison post-hoc tests were performed to understand the relationship between the numbers of whiteflies counted, uninfected cassava plants, infected cassava plants, and the mean of symptom severity in and across the States under investigation. Correlation coefficient matrix and the p-value computed using the field data are presented in Table 5 and Table 6 respectively. Table 7, Table 8, Table 9, Table 10, Table 11, Table 12, Table 13, Table 14 give the results of the ANOVA and multiple comparison post-hoc tests for whiteflies counted, uninfected cassava farms, infected cassava farms, and mean of symptom severity across the 12 States of Nigeria. Fig. 19, Fig. 20, Fig. 21, Fig. 22 show the mean comparisons of the four parameters for easy data interpretations.

**Table 5:** Correlation coefficient matrix.

	Whiteflies Counted	No. of uninfected farms	No. of infected farms	Mean of symptom severity
Whiteflies counted	1.0000	−0.0245	0.0225	0.1401
No. of uninfected plants	−0.0245	1.0000	−0.9985	−0.5853
No. of infected plants	0.0225	-0.9985	1.0000	0.5852
Mean of symptom severity	0.1401	-0.5853	0.5852	1.0000

**Table 6:** P-value matrix.

	Whiteflies counted	No. of uninfected farms	No. of infected farms	Mean of symptom severity
Whiteflies counted	1.0000	0.7419	0.7626	0.0585
No. of uninfected plants	0.7419	1.0000	0.0000	0.0000
No. of infected plants	0.7626	0.0000	1.0000	0.0000
Mean of symptom severity	0.0585	0.0000	0.0000	1.0000

**Table 7:** ANOVA test results for whiteflies counted in 184 farms in 12 Nigerian States.

Source of variation	Sum of squares	Degree of freedom	Mean squares	F statistic	Prob>F
Columns	3194.6	11	290.416	3.66	0.0001
Error	13584.8	171	79.444		
Total	16779.4	182

**Table 8:** Multiple comparison post-hoc test results for whiteflies counted in 184 farms in 12 Nigerian States.

Groups compared		Lower limits for 95% confidence intervals	Mean difference	Upper limits for 95% confidence intervals	*p*-value
Benue	Ekiti	−15.0550	−4.7879	5.4792	0.9343
Benue	Kogi	−8.3505	0.6667	9.6838	1.0000
Benue	Kwara	−17.0325	−7.0833	2.8658	0.4573
Benue	Lagos	−31.6379	−14.0000	3.6379	0.2829
Benue	Nassarawa	−10.1694	0.4667	11.1027	1.0000
Benue	Niger	−9.2360	0.4359	10.1078	1.0000
Benue	Ogun	−17.2730	−9.6190	−1.9651	0.0024
Benue	Ondo	−18.2111	−9.0000	0.2111	0.0627
Benue	Osun	−12.0325	−2.0833	7.8658	0.9999
Benue	Oyo	−10.9771	−3.0000	4.9771	0.9867
Benue	Plateau	−10.4037	0.6667	11.7370	1.0000
Ekiti	Kogi	−5.9542	5.4545	16.8633	0.9225
Ekiti	Kwara	−14.4542	-2.2955	9.8633	1.0000
Ekiti	Lagos	−28.1844	-9.2121	9.7601	0.9143
Ekiti	Nassarawa	−7.4724	5.2545	17.9815	0.9725
Ekiti	Niger	−6.7092	5.2238	17.1568	0.9577
Ekiti	Ogun	−15.1962	−4.8312	5.5338	0.9345
Ekiti	Ondo	−15.7748	−4.2121	7.3505	0.9898
Ekiti	Osun	−9.4542	2.7045	14.8633	0.9999
Ekiti	Oyo	−8.8179	1.7879	12.3937	1.0000
Ekiti	Plateau	−7.6376	5.4545	18.5466	0.9706
Kogi	Kwara	−18.8735	−7.7500	3.3735	0.4932
Kogi	Lagos	−32.9927	−14.6667	3.6594	0.2707
Kogi	Nassarawa	−11.9419	−0.2000	11.5419	1.0000
Kogi	Niger	−11.1070	−0.2308	10.6455	1.0000
Kogi	Ogun	−19.4142	−10.2857	−1.1572	0.0124
Kogi	Ondo	−20.1352	−9.6667	0.8019	0.1033
Kogi	Osun	−13.8735	−2.7500	8.3735	0.9997
Kogi	Oyo	−13.0677	−3.6667	5.7344	0.9823
Kogi	Plateau	−12.1367	0.0000	12.1367	1.0000
Kwara	Lagos	−25.7188	−6.9167	11.8854	0.9889
Kwara	Nassarawa	−4.9219	7.5500	20.0219	0.7084
Kwara	Niger	−4.1413	7.5192	19.1798	0.6175
Kwara	Ogun	−12.5859	−2.5357	7.5144	0.9996
Kwara	Ondo	−13.1979	−1.9167	9.3646	1.0000
Kwara	Osun	−6.8915	5.0000	16.8915	0.9685
Kwara	Oyo	−6.2150	4.0833	14.3817	0.9798
Kwara	Plateau	−5.0943	7.7500	20.5943	0.7128
Lagos	Nassarawa	−4.7078	14.4667	33.6411	0.3618
Lagos	Niger	−4.2210	14.4359	33.0928	0.3218
Lagos	Ogun	−13.3142	4.3810	22.0761	0.9997
Lagos	Ondo	−13.4222	5.0000	23.4222	0.9992
Lagos	Osun	−6.8854	11.9167	30.7188	0.6435
Lagos	Oyo	−6.8372	11.0000	28.8372	0.6830
Lagos	Plateau	−4.7521	14.6667	34.0854	0.3601
Nassarawa	Niger	−12.2827	−0.0308	12.2212	1.0000
Nassarawa	Ogun	−20.8163	−10.0857	0.6449	0.0891
Nassarawa	Ondo	−21.3582	−9.4667	2.4248	0.2785
Nassarawa	Osun	−15.0219	−2.5500	9.9219	1.0000
Nassarawa	Oyo	−14.4301	−3.4667	7.4967	0.9970
Nassarawa	Plateau	−13.1834	0.2000	13.5834	1.0000
Niger	Ogun	−19.8308	−10.0549	-0.2791	0.0373
Niger	Ondo	−20.4735	−9.4359	1.6017	0.1824
Niger	Osun	−14.1798	−2.5192	9.1413	0.9999
Niger	Oyo	−13.4667	−3.4359	6.5949	0.9939
Niger	Plateau	−12.4000	0.2308	12.8616	1.0000
Ogun	Ondo	−8.7011	0.6190	9.9392	1.0000
Ogun	Osun	−2.5144	7.5357	17.5859	0.3719
Ogun	Oyo	−1.4836	6.6190	14.7217	0.2415
Ogun	Plateau	−0.8755	10.2857	21.4470	0.1050
Ondo	Osun	−4.3646	6.9167	18.1979	0.6911
Ondo	Oyo	−3.5872	6.0000	15.5872	0.6621
Ondo	Plateau	−2.6148	9.6667	21.9481	0.2955
Osun	Oyo	−11.2150	−0.9167	9.3817	1.0000
Osun	Plateau	−10.0943	2.7500	15.5943	0.9999
Oyo	Plateau	−7.7186	3.6667	15.0519	0.9964

**Table 9:** ANOVA test results for number of uninfected cassava plants in 184 farms in 12 Nigerian States.

Source of variation	Sum of squares	Degree of freedom	Mean squares	F statistic	Prob>F
Columns	2178.73	11	198.067	3.46	0.0002
Error	9784.22	171	57.218		
Total	11962.95	182

**Table 10:** Multiple comparison post-hoc test results for number of uninfected cassava plants in 184 farms in 12 Nigerian States.

**Groups compared**		**Lower limits for 95% confidence intervals**	**Mean difference**	**Upper limits for 95% confidence intervals**	***p*-value**
Benue	Ekiti	−12.9254	−4.2121	4.5012	0.9167
Benue	Kogi	−16.5067	−8.8542	−1.2016	0.0086
Benue	Kwara	−11.6935	−3.2500	5.1935	0.9840
Benue	Lagos	−25.3020	−10.3333	4.6353	0.5085
Benue	Nassarawa	−17.5931	−8.5667	0.4598	0.0819
Benue	Niger	−14.5672	−6.3590	1.8493	0.3199
Benue	Ogun	−9.2694	−2.7738	3.7218	0.9647
Benue	Ondo	−12.6171	−4.8000	3.0171	0.6891
Benue	Osun	−15.4435	−7.0000	1.4435	0.2214
Benue	Oyo	−9.6865	−2.9167	3.8532	0.9623
Benue	Plateau	−22.8395	−13.4444	−4.0494	0.0002
Ekiti	Kogi	−14.3242	−4.6420	5.0401	0.9210
Ekiti	Kwara	−9.3566	0.9621	11.2808	1.0000
Ekiti	Lagos	−22.2223	−6.1212	9.9799	0.9855
Ekiti	Nassarawa	−15.1555	−4.3545	6.4464	0.9771
Ekiti	Niger	−12.2740	−2.1469	7.9802	0.9999
Ekiti	Ogun	−7.3581	1.4383	10.2347	1.0000
Ekiti	Ondo	−10.4007	−0.5879	9.2249	1.0000
Ekiti	Osun	−13.1066	−2.7879	7.5308	0.9993
Ekiti	Oyo	−7.7053	1.2955	10.2962	1.0000
Ekiti	Plateau	−20.3431	−9.2323	1.8785	0.2183
Kogi	Kwara	−3.8359	5.6042	15.0443	0.7339
Kogi	Lagos	−17.0318	−1.4792	14.0735	1.0000
Kogi	Nassarawa	−9.6774	0.2875	10.2524	1.0000
Kogi	Niger	−6.7351	2.4952	11.7255	0.9993
Kogi	Ogun	−1.6667	6.0804	13.8274	0.2998
Kogi	Ondo	−4.8301	4.0542	12.9385	0.9433
Kogi	Osun	−7.5859	1.8542	11.2943	1.0000
Kogi	Oyo	−2.0408	5.9375	13.9158	0.3841
Kogi	Plateau	−14.8903	−4.5903	5.7097	0.9520
Kwara	Lagos	−23.0400	−7.0833	8.8733	0.9533
Kwara	Nassarawa	−15.9011	−5.3167	5.2678	0.8938
Kwara	Niger	−13.0049	−3.1090	6.7869	0.9971
Kwara	Ogun	−8.0530	0.4762	9.0054	1.0000
Kwara	Ondo	−11.1240	−1.5500	8.0240	1.0000
Kwara	Osun	−13.8419	−3.7500	6.3419	0.9880
Kwara	Oyo	−8.4065	0.3333	9.0732	1.0000
Kwara	Plateau	−21.0949	−10.1944	0.7060	0.0929
Lagos	Nassarawa	−14.5060	1.7667	18.0393	1.0000
Lagos	Niger	−11.8591	3.9744	19.8078	0.9996
Lagos	Ogun	−7.4577	7.5595	22.5767	0.8923
Lagos	Ondo	−10.1009	5.5333	21.1676	0.9920
Lagos	Osun	−12.6233	3.3333	19.2900	0.9999
Lagos	Oyo	−7.7211	7.4167	22.5545	0.9092
Lagos	Plateau	−19.5911	−3.1111	13.3689	1.0000
Nassarawa	Niger	−8.1901	2.2077	12.6055	0.9999
Nassarawa	Ogun	−3.3138	5.7929	14.8995	0.6381
Nassarawa	Ondo	−6.3252	3.7667	13.8585	0.9875
Nassarawa	Osun	−9.0178	1.5667	12.1511	1.0000
Nassarawa	Oyo	−3.6543	5.6500	14.9543	0.7043
Nassarawa	Plateau	−16.2358	−4.8778	6.4803	0.9632
Niger	Ogun	−4.7112	3.5852	11.8815	0.9615
Niger	Ondo	−7.8082	1.5590	10.9262	1.0000
Niger	Osun	−10.5369	−0.6410	9.2549	1.0000
Niger	Oyo	−5.0705	3.4423	11.9551	0.9765
Niger	Plateau	−17.8048	−7.0855	3.6338	0.5789
Ogun	Ondo	−9.9358	−2.0262	5.8835	0.9996
Ogun	Osun	−12.7554	−4.2262	4.3030	0.9024
Ogun	Oyo	−7.0193	−0.1429	6.7336	1.0000
Ogun	Plateau	−20.1428	−10.6706	−1.1985	0.0124
Ondo	Osun	−11.7740	−2.2000	7.3740	0.9998
Ondo	Oyo	−6.2530	1.8833	10.0197	0.9998
Ondo	Plateau	−19.0673	−8.6444	1.7784	0.2208
Osun	Oyo	−4.6565	4.0833	12.8232	0.9335
Osun	Plateau	−17.3449	−6.4444	4.4560	0.7391
Oyo	Plateau	−20.1900	−10.5278	−0.8655	0.0191

**Table 11:** ANOVA test results for number of infected cassava plants in 184 farms in 12 Nigerian States.

Source of variation	Sum of squares	Degree of freedom	Mean squares	F statistic	Prob>F
Columns	2080.4	11	189.131	3.29	0.0004
Error	9817	171	57.409		
Total	11897.5	182

**Table 12:** Multiple comparison post-hoc test results for number of infected cassava plants in 184 farms in 12 Nigerian States.

Groups compared		Lower limits for 95% confidence intervals	Mean difference	Upper limits for 95% confidence intervals	*p*-value
Benue	Ekiti	−4.5158	4.2121	12.9400	0.9176
Benue	Kogi	1.1888	8.8542	16.5195	0.0088
Benue	Kwara	−5.2076	3.2500	11.7076	0.9843
Benue	Lagos	−4.6604	10.3333	25.3271	0.5112
Benue	Nassarawa	−0.4749	8.5667	17.6082	0.0831
Benue	Niger	−1.8630	6.3590	14.5809	0.3225
Benue	Ogun	−3.7327	2.7738	9.2803	0.9651
Benue	Ondo	−3.0302	4.8000	12.6302	0.6914
Benue	Osun	−1.4576	7.0000	15.4576	0.2236
Benue	Oyo	−3.9062	2.8750	9.6562	0.9665
Benue	Plateau	3.3670	12.7778	22.1885	0.0006
Ekiti	Kogi	−5.0563	4.6420	14.3404	0.9219
Ekiti	Kwara	−11.2981	−0.9621	9.3738	1.0000
Ekiti	Lagos	−10.0068	6.1212	22.2492	0.9857
Ekiti	Nassarawa	−6.4645	4.3545	15.1736	0.9774
Ekiti	Niger	−7.9972	2.1469	12.2909	0.9999
Ekiti	Ogun	−10.2494	−1.4383	7.3728	1.0000
Ekiti	Ondo	−9.2413	0.5879	10.4171	1.0000
Ekiti	Osun	−7.5481	2.7879	13.1238	0.9993
Ekiti	Oyo	−10.3530	−1.3371	7.6787	1.0000
Ekiti	Plateau	−2.5637	8.5657	19.6951	0.3301
Kogi	Kwara	−15.0601	−5.6042	3.8517	0.7360
Kogi	Lagos	−14.0995	1.4792	17.0578	1.0000
Kogi	Nassarawa	−10.2691	−0.2875	9.6941	1.0000
Kogi	Niger	−11.7409	−2.4952	6.7505	0.9993
Kogi	Ogun	−13.8404	−6.0804	1.6796	0.3024
Kogi	Ondo	−12.9533	−4.0542	4.8450	0.9440
Kogi	Osun	−11.3101	−1.8542	7.6017	1.0000
Kogi	Oyo	−13.9709	−5.9792	2.0125	0.3754
Kogi	Plateau	−6.3936	3.9236	14.2408	0.9855
Kwara	Lagos	−8.9000	7.0833	23.0667	0.9538
Kwara	Nassarawa	−5.2855	5.3167	15.9188	0.8949
Kwara	Niger	−6.8035	3.1090	13.0214	0.9972
Kwara	Ogun	−9.0197	−0.4762	8.0673	1.0000
Kwara	Ondo	−8.0400	1.5500	11.1400	1.0000
Kwara	Osun	−6.3588	3.7500	13.8588	0.9881
Kwara	Oyo	−9.1295	−0.3750	8.3795	1.0000
Kwara	Plateau	−1.3909	9.5278	20.4465	0.1587
Lagos	Nassarawa	−18.0666	−1.7667	14.5332	1.0000
Lagos	Niger	−19.8343	−3.9744	11.8856	0.9996
Lagos	Ogun	−22.6019	−7.5595	7.4828	0.8935
Lagos	Ondo	−21.1938	−5.5333	10.1271	0.9921
Lagos	Osun	−19.3167	−3.3333	12.6500	0.9999
Lagos	Oyo	−22.6215	−7.4583	7.7048	0.9069
Lagos	Plateau	−14.0631	2.4444	18.9520	1.0000
Nassarawa	Niger	−12.6229	−2.2077	8.2075	0.9999
Nassarawa	Ogun	−14.9148	−5.7929	3.3291	0.6406
Nassarawa	Ondo	−13.8754	−3.7667	6.3421	0.9877
Nassarawa	Osun	−12.1688	−1.5667	9.0355	1.0000
Nassarawa	Oyo	−15.0115	−5.6917	3.6282	0.6965
Nassarawa	Plateau	−7.1659	4.2111	15.5882	0.9884
Niger	Ogun	−11.8954	−3.5852	4.7251	0.9619
Niger	Ondo	−10.9418	−1.5590	7.8239	1.0000
Niger	Osun	−9.2714	0.6410	10.5535	1.0000
Niger	Oyo	−12.0110	−3.4840	5.0431	0.9746
Niger	Plateau	−4.3184	6.4188	17.1560	0.7250
Ogun	Ondo	−5.8967	2.0262	9.9491	0.9996
Ogun	Osun	−4.3173	4.2262	12.7697	0.9035
Ogun	Oyo	−6.7868	0.1012	6.9892	1.0000
Ogun	Plateau	0.5160	10.0040	19.4920	0.0283
Ondo	Osun	−7.3900	2.2000	11.7900	0.9999
Ondo	Oyo	−10.0750	−1.9250	6.2250	0.9998
Ondo	Plateau	−2.4625	7.9778	18.4181	0.3415
Osun	Oyo	−12.8795	−4.1250	4.6295	0.9296
Osun	Plateau	−5.1409	5.7778	16.6965	0.8550
Oyo	Plateau	0.2244	9.9028	19.5812	0.0394

**Table 13:** ANOVA test results for mean of Cassava mosaic diseases symptom severity.

Source of variation	Sum of squares	Degree of freedom	Mean squares	F statistic	Prob>F
Columns	14.223	11	1.293	1.91	0.0413
Error	115.911	171	0.67784		
Total	130.133	182

**Table 14:** Multiple comparison post-hoc test results for mean of Cassava mosaic disease symptom severity.

Groups compared		Lower limits for 95% confidence intervals	Mean difference	Upper limits for 95% confidence intervals	*p*-value
Benue	Ekiti	−1.3872	−0.4388	0.5096	0.9377
Benue	Kogi	−0.1585	0.6744	1.5073	0.2540
Benue	Kwara	−0.7682	0.1508	1.0698	1.0000
Benue	Lagos	−1.5426	0.0866	1.7159	1.0000
Benue	Nassarawa	−0.6881	0.2943	1.2768	0.9981
Benue	Niger	−0.5091	0.3843	1.2777	0.9627
Benue	Ogun	−0.5822	0.1248	0.8318	1.0000
Benue	Ondo	−0.5342	0.3166	1.1675	0.9878
Benue	Osun	−1.0382	−0.1192	0.7998	1.0000
Benue	Oyo	−0.4940	0.2429	0.9797	0.9956
Benue	Plateau	−0.2734	0.7492	1.7718	0.4098
Ekiti	Kogi	0.0594	1.1132	2.1670	0.0277
Ekiti	Kwara	−0.5335	0.5896	1.7127	0.8615
Ekiti	Lagos	−1.2270	0.5255	2.2779	0.9981
Ekiti	Nassarawa	−0.4424	0.7332	1.9088	0.6671
Ekiti	Niger	−0.2791	0.8231	1.9254	0.3784
Ekiti	Ogun	−0.3938	0.5637	1.5211	0.7443
Ekiti	Ondo	−0.3126	0.7555	1.8235	0.4682
Ekiti	Osun	−0.8035	0.3196	1.4427	0.9988
Ekiti	Oyo	−0.2980	0.6817	1.6614	0.4953
Ekiti	Plateau	−0.0213	1.1880	2.3973	0.0596
Kogi	Kwara	−1.5511	−0.5236	0.5039	0.8840
Kogi	Lagos	−2.2805	−0.5878	1.1050	0.9932
Kogi	Nassarawa	−1.4647	−0.3801	0.7046	0.9926
Kogi	Niger	−1.2947	−0.2901	0.7146	0.9987
Kogi	Ogun	−1.3927	−0.5495	0.2937	0.6011
Kogi	Ondo	−1.3247	−0.3578	0.6092	0.9884
Kogi	Osun	−1.8211	−0.7936	0.2339	0.3246
Kogi	Oyo	−1.2999	−0.4315	0.4369	0.9007
Kogi	Plateau	−1.0463	0.0748	1.1959	1.0000
Kwara	Lagos	−1.8009	−0.0642	1.6726	1.0000
Kwara	Nassarawa	−1.0085	0.1435	1.2956	1.0000
Kwara	Niger	−0.8436	0.2335	1.3106	0.9999
Kwara	Ogun	−0.9543	−0.0260	0.9024	1.0000
Kwara	Ondo	−0.8762	0.1658	1.2079	1.0000
Kwara	Osun	−1.3684	−0.2700	0.8284	0.9997
Kwara	Oyo	−0.8592	0.0921	1.0433	1.0000
Kwara	Plateau	−0.5880	0.5984	1.7848	0.8911
Lagos	Nassarawa	−1.5635	0.2077	1.9789	1.0000
Lagos	Niger	−1.4257	0.2977	2.0210	1.0000
Lagos	Ogun	−1.5963	0.0382	1.6727	1.0000
Lagos	Ondo	−1.4717	0.2300	1.9317	1.0000
Lagos	Osun	−1.9426	−0.2058	1.5309	1.0000
Lagos	Oyo	−1.4914	0.1563	1.8039	1.0000
Lagos	Plateau	−1.1312	0.6626	2.4563	0.9886
Nassarawa	Niger	−1.0417	0.0900	1.2217	1.0000
Nassarawa	Ogun	−1.1607	−0.1695	0.8217	1.0000
Nassarawa	Ondo	−1.0761	0.0223	1.1207	1.0000
Nassarawa	Osun	−1.5656	−0.4135	0.7385	0.9910
Nassarawa	Oyo	−1.0641	−0.0515	0.9612	1.0000
Nassarawa	Plateau	−0.7814	0.4549	1.6911	0.9889
Niger	Ogun	−1.1625	−0.2595	0.6435	0.9987
Niger	Ondo	−1.0872	−0.0677	0.9519	1.0000
Niger	Osun	−1.5806	−0.5035	0.5736	0.9332
Niger	Oyo	−1.0680	−0.1414	0.7851	1.0000
Niger	Plateau	−0.8019	0.3649	1.5316	0.9972
Ogun	Ondo	−0.6691	0.1918	1.0527	0.9999
Ogun	Osun	−1.1724	−0.2440	0.6843	0.9994
Ogun	Oyo	−0.6304	0.1180	0.8665	1.0000
Ogun	Plateau	−0.4066	0.6243	1.6553	0.7079
Ondo	Osun	−1.4779	−0.4358	0.6062	0.9697
Ondo	Oyo	−0.9593	−0.0738	0.8118	1.0000
Ondo	Plateau	−0.7019	0.4326	1.5670	0.9852
Osun	Oyo	−0.5892	0.3621	1.3133	0.9854
Osun	Plateau	−0.3180	0.8684	2.0548	0.4114
Oyo	Plateau	−0.5454	0.5063	1.5580	0.9189
