# Supplementary material for: Incidence of cassava mosaic disease and associated whitefly vectors in South West and North Central Nigeria: Data exploration
Source: Data Brief. 2018 May 19;19:370–92. doi: 10.1016/j.dib.2018.05.016 (PMC5993015; doi:10.1016/j.dib.2018.05.016)
Supplement: Supplementary file 1 — Supplementary material [file mmc1.docx]

**Conflict of Interest Form**

We confirm that there are no known conflicts of interest associated with this manuscript and that the manuscript has been read and approved by all named authors. We also confirm there has been no financial support for this work that could have influenced its outcome.
